# Associations between the quality of family interactions during a mother–father–adolescent conflict discussion task and physiological emotion regulation in adolescents

**DOI:** 10.1371/journal.pmen.0000246

**Published:** 2025-03-26

**Authors:** Michaël Romet, Nicolas Favez, Setareh Ranjbar, Sébastien Urben, Hervé Tissot

**Affiliations:** 1 Center for Family Studies, University Institute of Psychotherapy, Department of Psychiatry, Lausanne University Hospital and University of Lausanne, Lausanne, Switzerland; 2 Faculty of Psychology and Educational Sciences, University of Geneva, Geneva, Switzerland; 3 Center for Psychiatric Epidemiology and Psychopathology, Department of Psychiatry, Lausanne University Hospital and University of Lausanne, Lausanne, Switzerland; 4 Division of Child and Adolescent Psychiatry, Department of Psychiatry, Lausanne University Hospital and University of Lausanne, Lausanne, Switzerland; PLOS: Public Library of Science, UNITED KINGDOM OF GREAT BRITAIN AND NORTHERN IRELAND

## Abstract

Emotion regulation (ER) is a key competence in adolescence that is related to multiple psychological outcomes, including adaptive functioning and psychopathological symptoms. The development of ER skills is closely associated with a child’s family relational context. Nevertheless, few studies have investigated the physiological ER of adolescents during family interactions that go beyond the mother–adolescent dyad. Thus, little is understood regarding which features of communication in the mother–father–adolescent triad (e.g., warmth of affective exchanges) are relevant to adolescents’ physiological ER (as indexed by heart rate variability; HRV). The objective of this study was to explore the associations between the observed quality of triadic family interactions and adolescents’ physiological ER. This study investigated 77 mother–father–adolescent triads from the general population during a semi-standardized observational situation, the Lausanne Trilogue Play – Conflict Discussion Task (LTP–CDT). We assessed the quality of family interactions using an observational coding instrument, the Family Conflict and Alliance Assessment Scales – with adolescents (FCAAS), which includes scales on communicational aspects such as turn-taking, conflict resolution, affective climate, mentalization, and scales related to indicators of the coparenting relationship (e.g., coparenting support). We measured adolescents’ HRV before (baseline), during (reactivity), and after (recovery) the LTP–CDT by computing the root mean square of successive differences (RMSSD) between heartbeats as indices of adolescents’ physiological ER in each part of the procedure. Regression analyses showed significant associations between coparenting-related indicators (e.g., coparenting support) during mother–father–adolescent conflictual interactions and various measures of adolescents’ physiological ER capacities. These results highlight that family-level processes such as the coparenting relationship may be significant relational contexts for the socioemotional development of adolescents. The discussion encompasses research perspectives (e.g., extending our investigations to family triads involving a clinically referred member) and potential clinical implications (e.g., incorporating the coparenting unit into psychotherapy with adolescents).

## Introduction

Emotion regulation (ER) – the intra- and inter-personal capacity to manage one’s emotional reactions psychologically, behaviorally, and physiologically [1–3] – is fundamental for children’s and adolescents’ development [4]. Indeed, there is considerable evidence, mostly based on self-reports, that it is associated with many positive (e.g., adaptive functioning) and negative (e.g., psychopathology) outcomes [5–9]. At the physiological level, almost three decades of research [10] have linked ER to heart rate variability (HRV) or respiratory sinus arrhythmia (RSA), which are physiological indices of the balance between the sympathetic and the parasympathetic branches of the autonomic nervous system and, in particular, of the vagus nerve [3,11]. In laboratory situations mimicking daily life stressors, three different physiological processes of ER can be measured: pre-stressor (baseline resting state), during stressor (reactivity), and post-stressor (recovery resting state) [12]. Reactivity and recovery measures can be computed as baseline-to-task and task-to-resting differences. These processes inform about physiological regulation 1) in general or in the expectation of a stressor (i.e., baseline); 2) in reaction to a stressor (i.e., reactivity); 3) and when returning to calm or remaining in dysregulation after stress exposure (i.e., recovery). Measured with electrocardiograms, values of resting HRV have been associated with both positive (e.g., executive performances) and negative (e.g., conduct disorders) psychological outcomes [13–15], and values of HRV reactivity during social interactions have been shown to depend on whether interactions were positive (leading to HRV increase) or negative (leading to HRV decrease) [16,17]. Compared with self-reports, the line of research on interactions and physiology has brought much to the understanding of ER as it is a more objective way to measure ER that takes dynamic and situational aspects into account.

Aside from counting on their peers for support, adolescents still strongly rely on their parents [18] as they undergo numerous biological, cognitive, and affective changes [19,20]. A supportive parent–adolescent relationship, characterized by the parent’s emotional availability, acceptance, and adaptive communication, plays an essential role in the development of adolescents’ ER skills [21]. Much evidence suggests that observing family interactions is a valid and reliable method to evaluate the quality of family relationships [22–24]. Observation generally consists of the combination of a situation or laboratory task (e.g., a conflict discussion task; [25]) and a coding system that allows the assessment of specific behaviors (e.g., parental behaviors of promotion of adolescent autonomy; [26]) during videorecorded family interactions. Compared with questionnaires (i.e., measuring mental representations of family members about the family), fewer studies have used observation (i.e., measuring the quality of family interactions), although it provides an effective way to access family functioning in action, using a more objective approach based on several external viewpoints [27].

When interacting with one of the parents (researchers have mostly focused on dyadic mother–adolescent interactions; [28]), an adolescent will face numerous communication patterns and behaviors at both verbal and nonverbal levels [29,30]. For instance, the parent will speak warmly or behave with hostility [31], relate to the adolescent in a way that promotes or restricts their autonomy [31,32], mentalize—i.e., adequately verbalize and interpret the adolescent’s mental states—to various degrees [33,34], or validate the adolescent’s emotions and opinions to various degrees [35]. The parent will also express their own emotions or speak about emotions in general in various ways (e.g., as harmful and annoying or healthy and valid), and they may react to the adolescent’s emotions in a coaching or dismissing manner (i.e.,., emotion socialization and social learning theory [36–39]). These behaviors and many others may be either regulating or dysregulating for adolescents at the emotional [30,31,35,39–42] and physiological levels [43–51]. In particular, when in conflict, the quality of parent–adolescent communication may also be associated with adolescents’ emotional and physiological states during the discussion and play a significant role in their developmental outcomes [52–61].

The family relational context of adolescents, at least in two-parent families, includes a second parent, which forms a network of relationships that may play an important role in adolescents’ ER. More precisely, considering both parents will add several relevant family relationships (i.e., the relationship with each parent, marital relationship, coparenting relationship, mother–father–adolescent relationship) and levels in the family (i.e., dyadic and family levels). Beyond the parent–child dyad, family-level relational processes occur between the child(ren) and both parents and across dyadic relationships and within triadic and polyadic family relationships [62,63]. Amongst others, two family-level constructs have been investigated by researchers: coparenting (i.e., the degree of coordination, support, and conflict between the parents regarding childrearing [64]) and family alliance (FA; i.e., the degree of coordination in the mother–father–child triad [65,66]). These two constructs come from two different lines of research, and they are interconnected because the degree of coordination between coparents may affect the degree of coordination in the mother–father–child triad; however, coparenting and FA remain two separate constructs.

Regarding coparenting, its functioning will color emotionality and expressiveness in the family, as it has been shown to be associated with other family relationships and child and adolescent development [67–69]. For instance, coparenting is intimately linked to the marital relationship (the first directly concerns the child, whereas the second only directly concerns the romantic couple between the parents; [70]). Conflicts between parents (both in the coparenting and marital relationships) can easily spill over to each other and to other family relationships, such as the mother–child and father–child relationships [28,71,72]. According to the emotional security hypothesis, conflicts between the parents affect children’s and adolescents’ sense of emotional security in the family, which, in turn, will be linked to their adjustment [73–76]. In fact, it is not only the presence of conflicts between parents but the type of conflict that will influence children’s and adolescents’ feelings of security or insecurity within the family, as conflicts can be either constructive (e.g., involving collaborative behaviors aimed at resolution) or destructive (e.g., involving verbally and/or physically aggressive behaviors) [77,78]. In addition, self-reported coparenting conflict has also been associated with adolescents’ physiological ER (i.e., HRV) during mother–father–adolescent conflict discussions [79].

Regarding FA, investigations of the quality of mother–father–child interactions have been characterized based on verbal and nonverbal interactive behaviors such that three types of FA have been described: cooperative (functional), conflicted (dysfunctional), and disordered (dysfunctional). Cooperative FA refers to family interactions in which all members coordinate, co-construct, and share positive and authentic affects. Conflicted FA refers to family interactions in which overt or covert tensions between parents prevent coordination and positive affect sharing. Disordered FA refers to family interactions in which (self-)exclusion of a member (which may be caused by relational difficulties between parents) eliminates the possibility of coordination and positive affect sharing in the family triad. Most importantly, FA has been associated with other family relationships, such as marital, coparenting, and parent–child relationships [80–82], and it has been shown to predict psychological outcomes from infancy to adolescence [83–85]. Most recently, FA in infancy has also been shown to be specifically associated with infants’ physiological ER during mother–father–infant interactions [86].

The association between the quality of mother–father–adolescent interactions and adolescents’ ER has been little investigated at a behavioral level. When interacting with both parents simultaneously, an adolescent will deal with various relational processes and communication patterns at the verbal and nonverbal levels, which correspond to various degrees of coordination between coparents and in the mother–father–adolescent triad (i.e., coparenting and FA). For instance, parents could interrupt and undermine each other, and coparenting disagreements may be treated destructively (e.g., harsh criticism, raising of voice) so that the adolescent may be triangulated into the conflict, asked to take a stand for one parent, encouraged to criticize the other parent, or made guilty for the interparental conflict [87–90]. This lack of coordination between coparents and in the mother–father–adolescent triad may be disconcerting for adolescents, undermine their sense of emotional security in the family, and provoke emotional and physiological dysregulation [63,91]. Conversely, FA and coparenting may be functional, so that coparents will ideally be coordinated, support each other, and coparenting disagreements will be treated in a constructive manner (e.g., by calmly finding alternatives or compromise) so that the adolescent receives adapted, clear, and consistent signals or instructions from both parents and feels safe enough to interact and coordinate with parents even when undergoing family conflict [75,77]. Accordingly, numerous aspects such as coparenting and FA may hopefully provide a safe and joyful environment for adolescents’ daily learning experiences with emotions and their regulation, and positively affect their emotional development eventually [73,74,76,92–97].

One study investigated the association between mother–father–adolescent interactions and adolescents’ ER at the physiological level and suggested that the degree of cohesion and valence of affect sharing during triadic family interactions may be linked with late adolescents’ cortisol levels, another physiological indicator of ER skills, although this result was limited to males [98]. In another study, coparenting conflict (though it was measured using self-reports) was associated with adolescents’ HRV during mother–father–adolescent conflictual discussions [79], and adolescents’ HRV was associated with their sense of emotional security [76,99]. Consequently, these few studies may suggest that family-level processes during mother–father–adolescent interactions may be associated with adolescents’ physiological capacities and that these processes or specific features of mother–father–adolescent communication may act as protective or risk factors in the emotional development of adolescents.

Nevertheless, there are two main gaps. First, cortisol and HRV indices of physiological ER do not have the same response timing to stress, as HRV responds faster to stress (in a few seconds) and cortisol responds more slowly (in a few minutes) [100]. Because there is currently only one study measuring adolescents’ cortisol levels to associate them with the quality of observed triadic family interactions [98], there is a lack of knowledge about adolescents’ complete sequences of stress physiological responses to the quality of interactions, which could be addressed by measuring adolescents’ HRV. In addition, most current studies on physiology and family interactions have focused on baseline and reactivity measures of physiological regulation, while generally leaving recovery measures largely unexplored (e.g., [101]), although they also represent an important aspect of regulation [12]. Second, the other study used self-reports and did not assess coparenting during family interactions (although HRV was measured during mother–father–adolescent interactions), which did not allow to measure the association of the quality of these triadic interactions with adolescents’ HRV responses in real time [79]. Therefore, the relevance of the current study is that it investigates adolescent HRV measures (including recovery) in the context of triadic family interactions (including observed coparenting).

In conclusion, some evidence suggests that the quality of mother–father–adolescent interactions may be associated with adolescents’ HRV in response to these interactions; however, the literature is scarce. Therefore, the aim of the current study was to explore the association between the quality of triadic family interactions and adolescents’ physiological ER, as this is necessary to advance our understanding of the role of the family relational context on adolescents’ socio-emotional development. We adapted and extended the FA model to conflictual discussions in mother–father–adolescent triads and investigated FA, coparenting, and more specific features of mother–father–adolescent communication. We hypothesized that higher FA, better coparenting, and better communicational patterns during mother–father–adolescent conflictual interactions are associated with adolescents’ better physiological ER skills as indexed with HRV measures.

## Methods

### Participants

We recruited n = 78 mother–father–adolescent triads from the general population in Switzerland. Recruitment lasted from January 24, 2022, to January 31, 2023. Pediatricians and general practitioners were contacted to inquire whether flyers could be placed in their waiting rooms. The inclusion criteria were as follows: the adolescent was between 10 and 13 years old, and none of the participating family members had a known psychopathological diagnosis. Families were excluded from this study if parents were separated or divorced (n = 4), and one family was excluded because they came to the laboratory with a baby, the younger sibling, who cried during the family interaction and was thus brought to the mother in the middle of the task. The final sample was of n = 77 families and included 32 girls and 45 boys whose mean age was 12.04 years (*SD* =.92). The mothers’ mean age was 44.09 years (*SD* = 4.16; range: 34-60). The fathers’ mean age was 46.14 years (*SD* = 4.94; range: 33-59). Regarding the socioeconomic class of the families who participated, most were in the upper class (n = 53, 68.8%) according to the Swiss adaptation of Hollingshead’s two-factor index [102]. N = 3 families were in the lower-middle socioeconomic class (3.9%), n = 8 families were in the middle class (10.4%), and n = 13 families were in the upper-middle class (16.9%).

### Procedure

Approval regarding the research protocol was granted by Canton of Vaud’s ethics committee for research on humans (approval number: 2021-01859). Each parent signed an informed consent form regarding their participation and that of their adolescent. Adolescents were provided with a written explanation of the study in accessible language to inform them about the study and their right to consent or refuse participation. Adolescents’ consent was given verbally, as written consent is not necessary for minors under the age of 14 in the country of study. Families were invited to the laboratory to perform the Lausanne Trilogue Play – Conflict Discussion Task (LTP–CDT), a semi-standardized observational task eliciting conflictual interactions between the adolescent and the parents [103]. The design of this task was inspired by previous work on marital conflictual interactions and research on family triads using the Lausanne Trilogue Play [65,104,105]. The family triads were first instructed to select the most recent and tense conflictual topic for them. They were given examples, such as homework, screen time, and relationships between siblings. Once the topic was chosen, family members were instructed to attempt to solve this conflict in a 12-min discussion that followed a four-part scenario. First, one parent would discuss with the adolescent while the other parent remains simply present. Second, parents switch roles. Third, both parents discuss while the adolescent remains simply present. Finally, all members of the family triad discuss how to solve the family conflict. The start and transitions from one part to the next were signaled with clear and specific noise from an adjacent control room, so that each part lasted exactly 3 min. Once instructions were clear for family members, the family was left alone in the room to complete the task. This task was video-recorded using a two-camera setting so that, after data collection, researchers may code the quality of interactions using the video recordings.

In this sample, the order of the first two LTP–CDT parts was randomized: n = 38 families (49.4%) started the interaction in a mother–adolescent (+father) configuration, whereas n = 39 families (50.6%) started it in a father–adolescent (+mother) configuration. The conflictual topics selected by families in our sample included screen time (n = 23, 29.9% of the whole sample of families), school homework (n = 13, 16.9%), relationship with siblings or parental unfairness regarding treatment toward each sibling (n = 10, 13.0%), rule-setting and respect for rules in the family (n = 9, 11.7%), parent–adolescent relationship (n = 8, 10.4%), chores and cleaning at home (n = 5, 6.5%), adolescent autonomy (n = 3, 3.9%), family activities (n = 2, 2.6%), and other various topics (n = 4, 5.2%). Preliminary analyses showed that the variable of the conflictual subject discussed during LTP–CDT was not associated with our outcome measures (i.e., adolescents’ HRV).

Before, during, and after the LTP–CDT, HRV was measured in the adolescent as an index of their ER skills. The pre- and post-task electrocardiogram measures were taken in the resting state, which implies that the adolescents were seated comfortably with their eyes closed. They were instructed to move as little as possible and to remain silent for three minutes. At the end of the procedure, a short debriefing allowed family members to discuss the task and how it felt. Additionally, an optional video-feedback was given to families in the year following the encounter, which consisted of observing the video recording of interactions with our research team, who responded to parents’ questions and highlighted families’ resources [106].

#### Family alliance.

The Family Conflict and Alliance Assessment Scale – with adolescents (FCAAS) instrument is an observational coding system that was designed to assess the quality of mother–father–adolescent interactions, with a specific focus on FA and coparenting [103]. This observational tool was specifically designed to assess conflict interactions elicited during the LTP–CDT. It includes ten 5-point Likert scales: postures and gazes, turn-taking, mutual respect for coparenting roles, conflict resolution, affective climate, mentalization, role reversal, coparenting support, autonomy promotion, and adolescent autonomy (for a summary of FCAAS scales, see Table 1) [103]. Each scale represents a coherent ensemble of behavioral indicators related to a given construct that is considered relevant for assessing the quality of triadic family interactions. A score of 1 corresponds to “negative” or inappropriate family functioning, whereas a score of 5 corresponds to “positive” or appropriate family functioning. A recent validation study [107] demonstrated the validity and reliability of this instrument and suggested that the FCAAS has a two-factor structure: One factor is associated with the construct of FA, whereas the second factor is associated with the construct of coparenting. Each factor contains the scores of specific scales (i.e., mutual respect for coparenting roles, role reversal, and coparenting support for the coparenting factor; the remaining scales for the FA factor). Higher scores on the dimensions of FA and coparenting as well as on specific FCAAS scales are related to higher FA, better coparenting, and more functional communicational behaviors in the family. Therefore, this instrument allows for the observation of both larger constructs (i.e., FA and coparenting) and more specific constructs (e.g., conflict resolution).

**Table 1:** Summary of FCAAS scales.

Rating Scales	Brief description of the appropriate criteria
Postures & gazes	At the nonverbal level, participants signal their availability to interact, and everyone is involved in the interaction
Turn-taking	At the verbal level, everyone is engaged, talk time is balanced, and there are few interruptions or monologs
Mutual respect for coparenting roles	Both parents comply with the LTP–CDT instructions regarding interactive roles (third-party or active); they do not interfere with each other
Conflict resolution	Thanks to cooperation in the triad, a negotiation process allows the problem to be solved through dialog and co-construction of a viable solution
Affective climate	The climate is positive and warm, while affects are authentic; criticism is constructive and there is no attitude of defensiveness; family members seem to enjoy each other’s presence
Mentalization	Family members pay attention to their own and others’ mental states; they validate each other and there is a climate of empathy
Role reversal	There are neither triangulation nor coalitions between the parents or between one parent and the adolescent; no involvement of the adolescent in the parental subsystem
Coparenting support	Parents coordinate and support each other (whether there is agreement about education or not)
Autonomy promotion	Parents show respect for the adolescent’s individuality and help them identify and express their needs and preferences; limits are managed in a clear and flexible way
Adolescent autonomy	The adolescent demonstrates autonomy, independence, and self-approval

*Note*. The score ranges from 1 (inappropriate) to 5 (appropriate). Scores of 3 represent the moderate range. Scores of 2 and 4 allow nuances in the ratings.

Regarding the coding strategy, one rater (i.e., a member of the research team) coded all family interactions using the video recordings of these interactions, and n = 24 families (31.17% of the sample) were independently double coded by a second rater (i.e., another member of the research team). Estimation of inter-rater reliability followed the guidelines by Ten Hove and colleagues [108], which were developed to compute intraclass correlations (ICC) that consider the variance due to differing numbers of raters per subject. Based on their terminology, we calculated ICC (A, Khat) using the absolute agreement between raters, maximum likelihood estimation (MLE) of variance terms (i.e., lower bound, upper bound, and standard error), and the average ratings of the double-coded interactions in cases of disagreement. ICC coefficients were moderate to excellent for the 10 scales: postures and gazes (.71), turn-taking (.84), mutual respect for coparenting roles (.76), conflict resolution (.87), affective climate (.84), mentalization (.88), role reversal (.90), coparenting support (.86), autonomy promotion (.90), and adolescent autonomy (.81). There were no missing data.

#### Heart rate variability.

Cardiac activity of the adolescent was collected before, during, and after the LTP–CDT. Pre- and post-task electrocardiogram (ECG) allows baseline and recovery stress measurements, whereas ECG during the task allows stress reactivity measurements. Using HeartBIT BITalino ECG systems, ECG data were collected based on an Einthoven Lead II montage, and BITalino modules communicated in real time via Bluetooth with the OpenSignals (r)evolution software (v2.2.1) on a computer in the control room. The ECG signal was synchronized with the video data using OpenSignals software. Using Kubios HRV Premium (v3.5.0), the data were filtered, preprocessed, and analyzed so that HRV indicators were derived for the baseline resting state, LTP–CDT, and post-task resting. Kubios used a robust algorithm to correct peak artifacts and remove noise segments from the analyses [109,110]. Less than 1% of the beats on average were corrected in our dataset. Regarding noise, trained research assistants controlled for the automatically detected noise epochs and removed these epochs when appropriate, which resulted in approximately 1-2% of missing peak data. We particularly focused on the root mean square of successive differences (RMSSD), which represents a reliable HRV index [12]. High RMSSD or HRV occurs when the individual is relaxed and regulated at the emotional level, whereas low RMSSD or HRV occurs when the individual is perceiving an environmental threat and mobilizing a stress response [12]. Reactivity and recovery measures were computed as baseline-to-task and task-to-resting differences in RMSSD, respectively, so that higher values indicated higher stress reactivity and higher stress recovery. There were some missing HRV data: n = 2 for the task and reactivity measures and n = 4 for the recovery measure.

### Statistical analyses

First, we computed descriptive statistics (mean and standard deviation) and a set of Spearman coefficients of correlations between the investigated variables, including the age and sex of the adolescent as control variables. Second, to assess the associations between FCAAS dimensions (i.e., FA and coparenting) and adolescents’ physiological ER, we performed separate multiple linear regressions for each outcome measure of adolescents’ HRV (i.e., baseline, reactivity, and recovery). These regressions were serially adjusted as predictors were entered in blocks. The first block included the control variables, and the second block included the two dimensions of the FCAAS instrument. Third, to assess the associations between specific communication patterns during triadic family interactions and adolescents’ physiological ER, we performed the same analyses with a focus on FCAAS scales. In other words, we performed separate multiple linear regressions for each outcome measure of adolescents’ HRV, and regressions were serially adjusted. The first block included the control variables, the second block included FCAAS scales related to the FA dimension, and the third block included FCAAS scales related to the coparenting dimension. For all models, the estimated coefficients, standard errors, and standardized coefficients were reported. Using IBM SPSS 29.0, we performed statistical analyses and handled missing data with pairwise deletion.

## Results

Descriptive statistics and bivariate Spearman correlations are detailed in Table 2. Regarding the correlations, the FA dimension was significantly and positively correlated with the coparenting dimension and all FCAAS scales except for mutual respect for coparenting roles. Otherwise, FA also correlated significantly with adolescent sex, meaning that FA was higher in families with male adolescents. The coparenting dimension significantly and positively correlated with most scales except for postures & gazes and adolescent autonomy, and it correlated most strongly with the three scales related to this dimension (i.e., mutual respect for coparenting role, role reversal, coparenting support). The coparenting dimension was not significantly correlated with other study variables. FCAAS scales were significantly and strongly correlated with each other, but they did not correlate significantly with HRV measures. HRV measures were significantly and positively correlated with each other. Regarding control variables, the age of the adolescent was significantly and positively correlated with role reversal and with adolescent autonomy (i.e., older age was associated with less role reversal and more observed autonomy by the adolescent). Sex of the adolescent was significantly correlated with several variables, which indicated that in families of adolescent boys, there was significantly more conflict resolution, better affective climate, and parental promotion of adolescent autonomy. In addition, adolescent boys displayed a significantly higher baseline HRV than did adolescent girls.

**Table 2:** Spearman correlations and descriptive statistics of the study variables.

Study variables	n	1.	2.	3.	4.	5.	6.	7.	8.	9.	10.	11.	12.	13.	14.	15.	16.	17.
1. Family Alliance	77	1																
2. Coparenting	77	.50**	1															
3. Postures & Gazes	77	.48**	.09	1														
4. Turn-taking	77	.72**	.50**	.31**	1													
5. Mutual respect for coparenting roles	77	.11	.71**	−.13	.17	1												
6. Conflict resolution	77	.79**	.51**	.24*	.52**	.20	1											
7. Affective climate	77	.87**	.42**	.39**	.55**	.12	.69**	1										
8. Mentalization	77	.83**	.47**	.26*	.52**	.12	.59**	.66**	1									
9. Role reversal	77	.55**	.91**	.18	.48**	.50**	.54**	.43**	.51**	1								
10. Coparenting support	77	.53**	.83**	.14	.53**	.43**	.54**	.47**	.48**	.64**	1							
11. Auto promotion	77	.84**	.48**	.25*	.63**	.21	.67**	.70**	.73**	.50**	.47**	1						
12. Ado auto	77	.66**	.15	.29*	.32**	−.10	.43**	.53**	.50**	.25**	.17	.39**	1					
13. HRV baseline	76	.07	−.02	−.01	.03	−.08	.14	.06	.01	.07	−.07	.08	.08	1				
14. HRV reactivity	75	.08	.04	−.09	.13	−.07	.15	.05	−.01	.13	−.01	.20	−.02	.75**	1			
15. HRV recovery	73	.01	−.06	.02	.06	−.15	.07	−.01	−.04	.04	−.07	.11	−.10	.56**	.74**	1		
16. Ado age	73−77	.06	.15	−.05	.07	.08	.02	−.02	.04	.23*	.03	−.05	.24*	−.03	−.11	−.15	1	
17. Ado sex	73−77	.29*	.13	.08	.22	−.04	.36**	.22*	.20	.11	.21	.30**	.17	.26*	.21	.17	−.07	1
*Mean*		24.19	5.97	3.03	3.20	4.22	3.77	3.50	3.19	3.65	3.92	3.75	3.77	62.65	13.79	8.74	12.04	/
*SD*		11.79	2.97	0.99	1.01	0.95	1.04	1.21	1.27	1.35	1.20	1.21	1.23	34.99	19.66	19.26	0.92	/

*Note*. Auto = autonomy; Ado = adolescent; Ado sex: 0 = girls; 1 = boys. There were n = 45 boys (58%). * p <.05; ** p <.01.

Results of the multiple linear regression analyses are presented in Table 3 (predictors: FCAAS dimensions) and Table 4 (predictors: FCAAS scales). All parameters of the regression analyses (ANOVA tests, t-tests, exact p-values, 95% confidence intervals) can be found in S1 File. Regarding all the models using the two FCAAS dimensions, none of them were significantly associated with adolescents’ HRV measures; and none of the predictors were significant, except for adolescent sex. This coefficient indicated that compared with adolescent girls, adolescent boys showed higher HRV baseline values (both at steps 1 and 2).

**Table 3:** Multiple linear regression models using FCAAS dimensions to predict HRV in adolescents.

Variable	HRV baseline (n = 76)				HRV reactivity (n = 75)				HRV recovery (n = 73)			
	*B* (SE)	*β*	*R* ^*2*^	*ΔR* ^*2*^	*B* (SE)	*β*	*R* ^*2*^	*ΔR* ^*2*^	*B* (SE)	*β*	*R* ^*2*^	*ΔR* ^*2*^
Step 1			.07	.07			.04	.04			.03	.03
Ado age	−1.97 (4.28)	−0.05			−1.34 (2.47)	−0.06			−2.25 (2.46)	−0.11		
Ado sex	18.40* (7.97)	0.26*			7.06 (4.60)	0.18			5.05 (4.58)	0.13		
Step 2			.09	.02			.04	.00			.04	.01
Ado age	−1.34 (4.36)	−0.04			−1.27 (2.53)	−0.06			−2.01 (2.52)	−0.10		
Ado sex	19.75* (8.44)	0.28*			6.91 (4.90)	0.17			5.87 (4.88)	0.15		
FA dimension	0.14 (0.84)	0.02			0.20 (0.49)	0.06			−0.13 (0.48)	−0.04		
Coparenting dimension	−1.65 (1.63)	−0.14			−0.51 (0.95)	−0.08			−0.30 (0.94)	−0.05		

*Note*. Ado = adolescent; ado sex: 0 = girls; 1 = boys. *B* = estimated coefficients. SE = Standard error. β = estimated standardized coefficients. * *p* <.05. ** *p* <.01.

**Table 4:** Multiple linear regression models using FCAAS scales to predict HRV in adolescents.

Variable	HRV baseline (n = 76)				HRV reactivity (n = 75)				HRV recovery (n = 73)			
	*B* (SE)	β	*R* ^*2*^	*ΔR* ^*2*^	*B* (SE)	β	*R* ^*2*^	*ΔR* ^*2*^	*B* (SE)	β	*R* ^*2*^	*ΔR* ^*2*^
Step 1			.07	.07			.04	.04			.03	.03
Ado age	−1.97 (4.28)	−0.06			−1.34 (2.47)	−0.07			−2.25 (2.46)	−0.11		
Ado sex	18.40* (7.97)	0.26*			7.06 (4.60)	0.18			5.05 (4.58)	0.13		
Step 2			.11	.04			.10	.06			.07	.04
Ado age	−3.53 (4.74)	−0.10			−1.35 (2.70)	−0.07			−1.47 (2.72)	−0.07		
Ado sex	16.70 (8.95)	0.24			4.59 (5.11)	0.12			4.61 (5.15)	0.12		
Postures & gazes	−1.71 (4.67)	−0.05			−1.76 (2.66)	−0.09			0.09 (2.68)	0.00		
Turn-taking	0.38 (5.42)	0.01			1.87 (3.09)	0.10			1.73 (3.11)	0.09		
Conflict resolution	5.79 (6.07)	0.17			3.65 (3.47)	0.19			1.30 (3.49)	0.07		
Affective climate	−3.36 (5.65)	−0.12			−1.08 (3.23)	−0.07			−1.06 (3.25)	−0.07		
Mentalization	−4.51 (4.95)	−0.16			−3.52 (2.83)	−0.23			−2.98 (2.85)	−0.20		
Autonomy promotion	−0.53 (6.14)	−0.02			2.22 (3.50)	0.14			2.02 (3.53)	0.13		
Ado autonomy	3.95 (4.19)	0.14			−0.41 (2.39)	−0.03			−1.72 (2.41)	−0.11		
Step 3			.23	.11*			.24	.15*			.18	.11
Ado age	−6.24 (4.75)	−0.17			−2.94 (2.66)	−0.14			−2.89 (2.75)	−0.14		
Ado sex	18.03* (8.73)	0.26*			4.91 (4.89)	0.13			4.52 (5.05)	0.12		
Postures & gazes	−3.58 (4.59)	−0.10			−3.18 (2.57)	−0.16			−1.33 (2.65)	−0.07		
Turn-taking	2.84 (5.34)	0.08			3.24 (2.99)	0.17			2.56 (3.09)	0.14		
Conflict resolution	8.81 (6.37)	0.26			5.72 (3.57)	0.30			2.69 (3.68)	0.15		
Affective climate	−0.65 (5.50)	−0.02			0.47 (3.08)	0.03			0.10 (3.18)	0.01		
Mentalization	−3.83 (4.88)	−0.14			−3.30 (2.73)	−0.21			−3.14 (2.82)	−0.21		
Autonomy promotion	−3.39 (6.03)	−0.12			0.74 (3.38)	0.04			1.00 (3.49)	0.06		
Ado autonomy	2.61 (4.17)	0.09			−1.43 (2.33)	−0.09			−2.52 (2.41)	−0.16		
Mut respect coparenting roles	−7.20 (5.33)	−0.19			−6.01* (2.98)	−0.29*			−6.06 (3.08)	−0.30		
Role reversal	10.67* (5.10)	0.41*			6.78* (2.85)	0.47*			6.28* (2.95)	0.44*		
Coparenting support	−13.59* (5.26)	−0.47*			−7.70* (2.94)	−0.47*			−5.52 (3.04)	−0.35		

*Note*. Ado = adolescent; ado sex: 0 = girls; 1 = boys. Mut = mutual. *B* = estimated coefficients. SE = Standard error. β = estimated standardized coefficients. * *p* <.05. ** *p* <.01.

Regarding models that considered FCAAS scales as predictors of adolescents’ HRV, there were more significant associations. First, regarding adolescents’ baseline HRV, the models and predictors at steps 1 and 2 (control variables and FCAAS scales related to the FA factor, respectively) were non-significant, except for adolescent sex, which was significant at steps 1 and 3 of the analyses. This indicated that higher HRV baseline values were measured in adolescent boys than in adolescent girls. The addition of FCAAS scales related to coparenting at step 3 significantly improved the proportion of explained variance in the HRV baseline. A higher HRV baseline was positively associated with role reversal (i.e., less role reversal) and negatively associated with coparenting support (i.e., less coparenting support). Regarding the associations with adolescents’ HRV reactivity, the models and predictors at steps 1 and 2 were non-significant. The addition of FCAAS scales related to coparenting at step 3 significantly improved the proportion of explained variance in HRV reactivity. Higher HRV reactivity was significantly associated with lower degrees of mutual respect for coparenting roles, better scores on the role reversal scale (i.e., less role reversal), and less coparenting support. Regarding the associations with adolescents’ HRV recovery, the models and predictors at steps 1 and 2 did not explain a significant proportion of the variance in HRV recovery. At step 3, adding FCAAS scales related to the observed indicators of the coparenting relationship was not significant. Higher HRV recovery values were significantly associated with better scores at the scale of role reversal (i.e., less role reversal). For all final models (i.e., models including all blocks), the results of ANOVAs were not significant.

## Discussion

This study investigated the association between the quality of mother–father–adolescent interactions (i.e., FA, coparenting, and more specific communication patterns during triadic family interactions) and physiological ER skills in adolescents. The focus was on the adolescents’ HRV before, during, and after a conflictual family discussion (i.e., HRV baseline, reactivity, and recovery). The hypothesis was that higher FA, better coparenting, and better communicational patterns during mother–father–adolescent interactions are associated with better physiological ER in adolescents. The hypothesis was partially confirmed. Whereas the FA dimension, the coparenting dimension, and FA-related indicators were not significantly associated with adolescents’ HRV measures, this study highlighted that various specific aspects of the coparenting relationship observed during mother–father–adolescent conflictual discussions were associated with adolescents’ physiological ER capacities measured using HRV baseline, reactivity, and recovery. These results are discussed in light of previous findings and potential future research, and clinical implications.

Regarding the FA dimension of the FCAAS, it was not significantly associated with adolescents’ HRV baseline, reactivity, and recovery, which suggests that our analyses did not underline an association between the degree of coordination in mother–father–adolescent triads and adolescent’s physiological ER. Similarly, a deeper look at the FCAAS scales related to FA did not underline any significant association. To assess these results, there are no available studies of reference on the specific association between FA or the quality of triadic family interactions and adolescents’ HRV; however, a few studies assessing associations close to ours can be mentioned as some of them do not align with our results.

Indeed, our results do not converge with the study by Gans & Johnson [98], who reported a significant association between the quality of collaborative interactions in mother–father–adolescent triads and adolescents’ cortisol levels; however, this could suggest that the association between the quality of triadic family interactions and adolescents’ physiological ER may depend on the type of interaction (i.e., conflictual or collaborative) or physiological stress system (i.e., autonomic nervous system or hypothalamic–pituitary–adrenal axis). Also, there is evidence from research on early families suggesting that the quality of mother–father–infant or parent–infant interactions are associated with infants’ physiological ER as indexed with HRV measures [86,101]; however, the difference with our results on adolescence may suggest that the quality of family interactions might be associated in a smaller magnitude or in a different way with physiological ER in adolescents than in infancy, as ER evolves more and more from coregulation to self-regulation across development [111]. In addition, previous literature on parent–adolescent dyadic interactions seems more contrasted, as some of them report links with RSA reactivity [56,58], whereas other studies do not find such links [60,61]. This mixed evidence from dyadic research could indicate that the bivariate association between the quality of dyadic interactions and adolescents’ physiological ER might be moderated by other variables, whether it be psychopathological symptoms of one of the parents [112], of the adolescent [57], the degree of stress in the daily life of the adolescent [113], or other variables. In the case of the association between FA (or indicators related to this dimension) and adolescents’ physiological ER, it may also be moderated by other variables in a similar way (e.g., clinical status of one of the parents). Finally, our lack of significant results regarding FA or related behavioral indicators may be due to our sample of well-functioning families (i.e., see the high mean scores on the FCAAS scales in Table 2), whose conflicts may not have been intense enough to provoke strong physiological reactions in adolescents (or more between-subject variance in their reactions). Indeed, families displaying lower degrees of adaptive communication may function with relational dynamics that may be potentially more stressful and physiologically activating for adolescents. Accordingly, it would be interesting for future research to investigate our association of interest with clinical families and the inclusion of additional moderating variables, as this could provide a more complete picture of the potential role of family-level relational processes in the development of ER skills in adolescents.

Regarding the coparenting dimension of the FCAAS instrument, it was not significantly associated with physiological ER in adolescents; however, the specific behavioral indicators of this dimension appeared to be significantly associated with some measures of adolescent HRV. First, mutual respect for coparenting roles was negatively associated with HRV reactivity. This negative association could be expected because it suggests that when coparents respect each other, for instance, by not interfering in the interaction between the other parent and the adolescent (i.e., part of the LPT-CDT), adolescents’ HRV reactivity is reduced, and they experience lower levels of physiological stress.

Second, there was a positive association between the role reversal scale and all three measures of HRV, which indicated that when family interactions were characterized by clearer intergenerational boundaries or when adolescents were in the role of the child rather than that of the adult, adolescents experienced significantly less physiological stress before the interaction, more stress reactivity (during the interaction), and more stress recovery or better capacity to return to calm (after the interaction). To the best of our knowledge, there is no available evidence on this specific association, except for studies that have shown more general associations between family alliances and coalitions (indicators of role reversal) and psychological outcomes [90,114], which may in turn be related to emotional development and ER. At the relational level, this may suggest that when family hierarchy places the adolescent in the position of the child, the adolescent might feel generally more regulated (baseline and recovery measures), but during conflict interactions, they might also be dysregulated because they feel less in control of the turn of events regarding the conflict theme (screen time, family activities or other things) or even feel restricted in their need for increasing autonomy (an adolescent may often desire to be considered an adult or to reach the position of the adult). Another interesting idea is that mild and normative levels of role reversal may induce some stress-relieving emotional proximity between the adolescent and the parent(s), although this was only observed during resting measures pre- and post-interaction (as the adolescent prepares for the conflict and as they get out of it), which would then pose the question of how such an effect was not present during the interaction itself. One additional interpretation could be made by inverting the usual causal direction according to which the quality of family interactions influences adolescents’ physiological ER, and to consider that adolescents’ stress may influence the quality of family interactions. This suggests that when adolescents were more physiologically dysregulated, family interactions were characterized by clearer intergenerational boundaries, which would indicate that families may have reacted to adolescents’ dysregulation by attempting to provide a safer, clearer relational environment. Accordingly, such an interpretation would support a circularity or reciprocal influence between the family and the child, which is consistent with a systemic approach of the family [115].

Third, there was a significant negative association between observed coparenting support and adolescents’ HRV baseline and reactivity, which indicates that when the coparenting relationship is characterized by more support, adolescents were more dysregulated during the baseline measure and less reactive to the stressful condition of discussing a heated conflict with both parents at the same time. Although this may seem surprising at first, it may indicate that adolescents may have felt more stress or needed more physiological resources when preparing to face a strong and unified coparenting team. For instance, an adolescent aiming to receive additional daily screen time might be more likely to achieve this goal during the following discussion if parents disagree or are unclear regarding the educational rules they want to implement, which may leave more room for the adolescents’ arguments and viewpoints. The expected impossibility of getting their way in the argument may have left the adolescent emotionally dysregulated because of the perspective of not being able to achieve more autonomy. It is possible that the adolescent experiencing more agreeable interactions with a coordinated coparenting team may have ended less reactive and more relaxed during the task, contrary to their expectations during the resting baseline measure. Finally, the abovementioned idea of circularity between the family and the child could also be reported here, and it would suggest that when adolescents were physiologically dysregulated during the resting baseline, parents may have reacted by showing more support to each other to provide a good-enough context to help adolescents regulate their emotions.

Finally, our findings on coparenting-related aspects of mother–father–adolescent interactions suggest that family-level processes may be associated with physiological ER capacities in adolescents. These results echo those of a previous study on the association between adolescent HRV and coparenting, although this study was limited to a self-reported measure of coparenting conflict [79]. In addition, our results align more generally with the literature supporting links between coparenting and adolescent outcomes [67,68,116]. Our findings extend this association to an observational measure of various domains or indicators of coparenting (and not only coparenting conflict). Further investigations are needed to deepen our understanding of the underlying mechanisms that may explain how only specific aspects of the coparenting relationship during conflictual family interactions are associated with specific aspects of physiological ER in adolescents.

Regarding the three HRV measures, as presented in the introduction, their use was helpful in collecting data on the three processes of physiological ER; that is, physiological regulation pre-task (i.e., baseline), during task (i.e., reactivity), and post-task (i.e., recovery) [12]. Our results suggested slightly more frequent significant associations between observed indicators of coparenting and reactivity measures of adolescent HRV, whereas associations with baseline and recovery measures seemed slightly less frequent but still present. These results indicate that the quality of triadic family interactions may not only affect adolescents’ physiological ER during interactions but also in the preceding and the following moments, although these effects could be weaker. Indeed, during the baseline phase, adolescents who anticipate destructive conflictual interactions with their parents may regulate themselves differently than those who expect constructive conflictual interactions [77]. Similarly, during the recovery phase, the quality of the preceding interactions can either facilitate or hinder adolescents’ ability to recuperate as quickly and efficiently as possible (i.e., returning to a state close to baseline). Therefore, our results support the investigation of physiological ER along the various processes surrounding a stressor (i.e., baseline, reactivity, and recovery) [12], and future studies could also explore the idea of computing general scores of physiological ER that combine the three physiological processes.

Regarding control variables, adolescent age did not appear to play a significant role in predicting HRV and was not associated with the quality of family interactions, except for the scales of role reversal and adolescent autonomy, which suggested that older age was associated with more adolescent autonomy and with less role reversal. The first correlation seems intuitive as adolescent development is characterized by increasing autonomy, and the second correlation is surprising, but it could suggest that as adolescents grow older, parents who involve the child in the parental subsystem become more and more able to abstain, or it could also mean that role reversal may be less and less visible by coders as adolescents get closer to the position of an adult. Regarding adolescent sex, it was surprising to see that higher FA, better conflict resolution, and affective climate were observed in families with male adolescents, which should be further investigated to reach a better understanding of the family’s role in adolescents’ ER according to their sex. The association showing that there was more parental promotion of autonomy for male adolescents could be due to gender-differentiated parenting, although this literature is contrasted and does not highlight large effects [117]. In addition, adolescent sex was significantly associated with the HRV resting baseline measure (and not with reactivity and recovery HRV measures). Such an association between sex and HRV echoes the literature, which shows that adolescent males typically display higher HRV values [118]. Accordingly, future studies investigating the associations between the quality of triadic family interactions and the physiological ER of adolescents should at least include adolescent sex as a control variable and could also explore its moderating effect.

There are two main limitations to our study. First, the small sample size prevented us from having sufficient statistical power to detect small- to medium-sized effects. Therefore, more studies should be conducted with larger samples, which could also allow testing for additional control variables such as the adolescent’s reported symptoms, the family’s socioeconomic status, or parents’ marital satisfaction. Second, our sample was mostly limited to families with two heterosexual parents from the middle-upper and upper classes, which came from the general population in Switzerland. Therefore, interpretations and conclusions from our results should be cautiously limited to traditional families, and we recommend that future studies extend research to families with low-income, same-sex parents, and even to clinically referred families.

Despite these limitations, the present study ventures into relatively unknown territory to investigate which family-level processes such as FA and coparenting, including more specific communicational behaviors in mother–father–adolescent triads, are associated with adolescents’ physiological ER skills measured through HRV. Therefore, the strengths of this study are that it explores the larger family relational context of adolescents (i.e., family processes beyond the parent–adolescent dyad) and that it combines observational and physiological methods to assess the association between family and emotional processes. Our findings only highlight some associations between the quality of mother–father–adolescent interactions and adolescents’ HRV, which may suggest an absence of association or, on the contrary, that this association may not be bivariate but more complex as it may interact with other variables. Nevertheless, looking at coparenting-specific aspects of communication during mother–father–adolescent interactions, our analyses underlined several important associations between observed coparenting and adolescents’ HRV, thereby supporting not only that the coparenting relationship plays a critical role in the mother–father–adolescent relationship but also that coparenting specifically might be an important factor for adolescent development in terms of ER and more general psychological outcomes.

With regard to clinical implications, these findings suggest that clinical work with adolescents might benefit from including not only mothers but also fathers to consider the coparenting and mother–father–adolescent relationships as potentially relevant risk or protective factors in the emotional and psychological development of adolescents. Accordingly, our findings could provide new avenues for prevention and intervention targeting developmental psychopathology. With regard to research perspectives, we hope that our study will foster more research combining observational and physiological methodologies and more research on the mother–father–adolescent triad as relevant alternatives to investigate the links between the quality of family relationships, adolescents’ ER, and psychological outcomes. Future studies should focus on other populations, such as low-income or clinically referred families.

## Supporting information

S1 File All parameters of all regression analyses. (PDF)

S2 File Excel dataset of the study variables. (XLSX)
