# Supplementary material for: Associations between the quality of family interactions during a mother–father–adolescent conflict discussion task and physiological emotion regulation in adolescents
Source: PLOS Ment Health. 2025 Mar 26;2(3):e0000246. doi: 10.1371/journal.pmen.0000246 (PMC12798475; doi:10.1371/journal.pmen.0000246)
Supplement: S1 File — (PDF) [file pmen.0000246.s001.pdf]

Supplementary material: SPSS outputs

Multiple linear regression 1

Predictors: FCAAS dimensions (+ control variables: age and sex of adolescent)

Outcome: HRV_baseline

Variables Entered/Removed[a]

Model	Variables Entered	Variables Removed	Method
1	Ado_sex, Ado_age[b]	.	Enter
2	FCAAS_coparenting_dimension, FCAAS_Family_Alliance_dimension[b]	.	Enter

a. Dependent Variable: HRV_baseline

b. All requested variables entered.

Model Summary

Model	R	R Square	Adjusted R Square	Std. Error of the Estimate	Change Statistics				
					R Square Change	F Change	df1	df2	Sig. F Change
1	,270[a]	,073	,048	34,14550	,073	2,871	2	73	,063
2	,298[b]	,089	,037	34,32568	,016	,618	2	71	,542

a. Predictors: (Constant), Ado_sex, Ado_age

b. Predictors: (Constant), Ado_sex, Ado_age, FCAAS_coparenting_dimension, FCAAS_Family_Alliance_dimension

ANOVA[a]

Model		Sum of Squares	df	Mean Square	F	Sig.
1	Regression	6694,952	2	3347,476	2,871	,063[b]
	Residual	85111,813	73	1165,915		
	Total	91806,765	75			
2	Regression	8150,831	4	2037,708	1,729	,153[c]
	Residual	83655,934	71	1178,253		
	Total	91806,765	75			

a. Dependent Variable: HRV_baseline

b. Predictors: (Constant), Ado_sex, Ado_age

c. Predictors: (Constant), Ado_sex, Ado_age, FCAAS_coparenting_dimension, FCAAS_Family_Alliance_dimension

Coefficients[a]

Model		Unstandardized Coefficients		Standardized Coefficients	t	Sig.	95.0% Confidence Interval for B		Collinearity Statistics	
		B	Std. Error	Beta			Lower Bound	Upper Bound	Tolerance	VIF
1	(Constant)	75,623	52,257		1,447	,152	-28,524	179,770		
	Ado_age	-1,971	4,281	-,052	-,460	,647	-10,502	6,561	,994	1,006
	Ado_sex	18,402	7,973	,261	2,308	,024	2,511	34,292	,994	1,006
2	(Constant)	83,391	53,349		1,563	,122	-22,983	189,765		
	Ado_age	-1,341	4,357	-,035	-,308	,759	-10,029	7,347	,969	1,032
	Ado_sex	19,746	8,437	,280	2,340	,022	2,923	36,569	,897	1,115
	FCAAS_Family_Alliance_dimension	,139	,836	,024	,166	,868	-1,529	1,807	,630	1,587
	FCAAS_coparenting_dimension	-1,654	1,632	-,140	-1,014	,314	-4,907	1,600	,670	1,493

a. Dependent Variable: HRV_baseline

Multiple linear regression 2

Predictors: FCAAS dimensions (+ control variables: age and sex of adolescent)

Outcome: HRV_reactivity

Variables Entered/Removed[a]

Model	Variables Entered	Variables Removed	Method
1	Ado_sex, Ado_age[b]	.	Enter
2	FCAAS_coparenting_dimension, FCAAS_Family_Alliance_dimension[b]	.	Enter

a. Dependent Variable: HRV_reactivity

b. All requested variables entered.

Model Summary

					Change Statistics				
Model	R	R Square	Adjusted R Square	Std. Error of the Estimate	R Square Change	F Change	df1	df2	Sig. F Change
1	,194[a]	,037	,011	19,55872	,037	1,401	2	72	,253
2	,204[b]	,042	-,013	19,79250	,004	,155	2	70	,857

a. Predictors: (Constant), Ado_sex, Ado_age

b. Predictors: (Constant), Ado_sex, Ado_age, FCAAS_coparenting_dimension, FCAAS_Family_Alliance_dimension

ANOVA[a]

Model		Sum of Squares	df	Mean Square	F	Sig.
1	Regression	1071,693	2	535,847	1,401	,253[b]
	Residual	27543,142	72	382,544		
	Total	28614,836	74			
2	Regression	1192,826	4	298,206	,761	,554[c]
	Residual	27422,010	70	391,743		
	Total	28614,836	74			

a. Dependent Variable: HRV_reactivity

b. Predictors: (Constant), Ado_sex, Ado_age

c. Predictors: (Constant), Ado_sex, Ado_age, FCAAS_coparenting_dimension, FCAAS_Family_Alliance_dimension

Coefficients[a]

		Unstandardized Coefficients		Standardized Coefficients			95.0% Confidence Interval for B		Collinearity Statistics	
Model		B	Std. Error	Beta	t	Sig.	Lower Bound	Upper Bound	Tolerance	VIF
1	(Constant)	25,814	30,134		,857	,394	-34,258	85,886		
	Ado_age	-1,341	2,468	-,063	-,543	,589	-6,262	3,579	,994	1,006
	Ado_sex	7,059	4,598	,178	1,535	,129	-2,107	16,224	,994	1,006
2	(Constant)	26,398	30,968		,852	,397	-35,367	88,162		
	Ado_age	-1,272	2,529	-,060	-,503	,617	-6,316	3,773	,969	1,032
	Ado_sex	6,908	4,898	,174	1,410	,163	-2,860	16,676	,897	1,115
	FCAAS_Family_Alliance_dimension	,195	,486	,059	,401	,690	-,774	1,163	,630	1,587
	FCAAS_coparenting_dimension	-,513	,947	-,077	-,541	,590	-2,402	1,376	,670	1,493

a. Dependent Variable: HRV_reactivity

Multiple linear regression 3

Predictors: FCAAS dimensions (+ control variables: age and sex of adolescent)

Outcome: HRV_recovery

Variables Entered/Removed[a]

Model	Variables Entered	Variables Removed	Method
1	Ado_sex, Ado_age[b]	.	Enter
2	FCAAS_coparenting_dimension, FCAAS_Family_Alliance_dimension[b]	.	Enter

a. Dependent Variable: HRV_recovery

b. All requested variables entered.

Model Summary

Model	R	R Square	Adjusted R Square	Std. Error of the Estimate	Change Statistics				
					R Square Change	F Change	df1	df2	Sig. F Change
1	,176[a]	,031	,003	19,22880	,031	1,114	2	70	,334
2	,190[b]	,036	-,021	19,45611	,005	,187	2	68	,830

a. Predictors: (Constant), Ado_sex, Ado_age

b. Predictors: (Constant), Ado_sex, Ado_age, FCAAS_coparenting_dimension, FCAAS_Family_Alliance_dimension

ANOVA[a]

Model		Sum of Squares	df	Mean Square	F	Sig.
1	Regression	823,448	2	411,724	1,114	,334[b]
	Residual	25882,269	70	369,747		
	Total	26705,718	72			
2	Regression	964,976	4	241,244	,637	,638[c]
	Residual	25740,742	68	378,540		
	Total	26705,718	72			

a. Dependent Variable: HRV_recovery

b. Predictors: (Constant), Ado_sex, Ado_age

c. Predictors: (Constant), Ado_sex, Ado_age, FCAAS_coparenting_dimension, FCAAS_Family_Alliance_dimension

Coefficients[a]

Model		Unstandardized Coefficients		Standardized Coefficients	t	Sig.	95.0% Confidence Interval for B		Collinearity Statistics	
		B	Std. Error	Beta			Lower Bound	Upper Bound	Tolerance	VIF
1	(Constant)	32,924	30,035		1,096	,277	-26,979	92,826		
	Ado_age	-2,254	2,460	-,108	-,916	,363	-7,161	2,653	,994	1,006
	Ado_sex	5,050	4,583	,130	1,102	,274	-4,090	14,189	,994	1,006
2	(Constant)	36,191	30,862		1,173	,245	-25,393	97,775		
	Ado_age	-2,012	2,521	-,097	-,798	,427	-7,042	3,018	,969	1,032
	Ado_sex	5,873	4,881	,151	1,203	,233	-3,867	15,613	,897	1,115
	FCAAS_Family_Alliance_dimension	-,130	,484	-,040	-,269	,788	-1,096	,835	,630	1,587
	FCAAS_coparenting_dimension	-,297	,944	-,046	-,315	,754	-2,181	1,586	,670	1,493

a. Dependent Variable: HRV_recovery

Multiple linear regression 4

Predictors: FCAAS scales (+ control variables: age and sex of adolescent)

Outcome: HRV_baseline

Variables Entered/Removed[a]

Model	Variables Entered	Variables Removed	Method
1	Ado_sex, Ado_age[b]	.	Enter
2	Postures_gazes, Mentalization, Adolescent_autonomy, Turn_taking, Conflict_resolution, Affective_climate, Autonomy_promotion[b]	.	Enter
3	Mutual_respect_for_coparenting_roles, Coparenting_support, Role_reversal[b]	.	Enter

a. Dependent Variable: HRV_baseline

b. All requested variables entered.

Model Summary

Model	R	R Square	Adjusted R Square	Std. Error of the Estimate	Change Statistics				
					R Square Change	F Change	df1	df2	Sig. F Change
1	,270[a]	,073	,048	34,14550	,073	2,871	2	73	,063
2	,331[b]	,110	-,012	35,19130	,037	,389	7	66	,905
3	,472[c]	,223	,075	33,64986	,113	3,062	3	63	,034

a. Predictors: (Constant), Ado_sex, Ado_age

b. Predictors: (Constant), Ado_sex, Ado_age, Postures_gazes, Mentalization, Adolescent_autonomy, Turn_taking, Conflict_resolution, Affective_climate, Autonomy_promotion

c. Predictors: (Constant), Ado_sex, Ado_age, Postures_gazes, Mentalization, Adolescent_autonomy, Turn_taking, Conflict_resolution, Affective_climate, Autonomy_promotion, Mutual_respect_for_coparenting_roles, Coparenting_support, Role_reversal

ANOVA[a]

Model		Sum of Squares	df	Mean Square	F	Sig.
1	Regression	6694,952	2	3347,476	2,871	,063[b]
	Residual	85111,813	73	1165,915		
	Total	91806,765	75			
2	Regression	10070,548	9	1118,950	,904	,528[c]
	Residual	81736,216	66	1238,428		
	Total	91806,765	75			
3	Regression	20471,047	12	1705,921	1,507	,146[d]
	Residual	71335,718	63	1132,313		
	Total	91806,765	75			

a. Dependent Variable: HRV_baseline

b. Predictors: (Constant), Ado_sex, Ado_age

c. Predictors: (Constant), Ado_sex, Ado_age, Postures_gazes, Mentalization, Adolescent_autonomy, Turn_taking, Conflict_resolution, Affective_climate, Autonomy_promotion

d. Predictors: (Constant), Ado_sex, Ado_age, Postures_gazes, Mentalization, Adolescent_autonomy, Turn_taking, Conflict_resolution, Affective_climate, Autonomy_promotion, Mutual_respect_for_coparenting_roles, Coparenting_support, Role_reversal

Coefficients[a]

Model		Unstandardized Coefficients		Standardized Coefficients	t	Sig.	95.0% Confidence Interval for B		Collinearity Statistics	
		B	Std. Error	Beta			Lower Bound	Upper Bound	Tolerance	VIF
1	(Constant)	75,623	52,257		1,447	,152	-28,524	179,770		
	Ado_age	-1,971	4,281	-,052	-,460	,647	-10,502	6,561	,994	1,006
	Ado_sex	18,402	7,973	,261	2,308	,024	2,511	34,292	,994	1,006
2	(Constant)	90,749	57,413		1,581	,119	-23,880	205,377		
	Ado_age	-3,526	4,735	-,093	-,745	,459	-12,979	5,927	,863	1,159
	Ado_sex	16,704	8,952	,237	1,866	,067	-1,170	34,577	,837	1,194
	Postures_gazes	-1,706	4,666	-,048	-,366	,716	-11,022	7,611	,779	1,283
	Turn_taking	,376	5,415	,011	,069	,945	-10,436	11,188	,548	1,825
	Conflict_resolution	5,786	6,072	,173	,953	,344	-6,337	17,908	,411	2,432
	Affective_climate	-3,356	5,652	-,116	-,594	,555	-14,641	7,929	,352	2,839
	Mentalization	-4,505	4,950	-,163	-,910	,366	-14,389	5,378	,418	2,393
	Autonomy_promotion	-,530	6,136	-,018	-,086	,931	-12,781	11,720	,298	3,354
	Adolescent_autonomy	3,947	4,185	,139	,943	,349	-4,409	12,303	,621	1,609
3	(Constant)	157,981	60,830		2,597	,012	36,421	279,540		
	Ado_age	-6,242	4,749	-,165	-1,314	,194	-15,732	3,249	,784	1,275
	Ado_sex	18,033	8,729	,256	2,066	,043	,590	35,476	,805	1,242
	Postures_gazes	-3,580	4,589	-,101	-,780	,438	-12,750	5,591	,737	1,357
	Turn_taking	2,839	5,342	,082	,532	,597	-7,835	13,514	,515	1,942
	Conflict_resolution	8,811	6,371	,263	1,383	,172	-3,921	21,542	,341	2,929
	Affective_climate	-,654	5,498	-,023	-,119	,906	-11,641	10,332	,340	2,937
	Mentalization	-3,834	4,876	-,139	-,786	,435	-13,578	5,910	,394	2,539
	Autonomy_promotion	-3,385	6,029	-,117	-,561	,576	-15,434	8,664	,282	3,543
	Adolescent_autonomy	2,608	4,166	,092	,626	,534	-5,717	10,934	,573	1,744
	Mutual_respect_for_coparenting_roles	-7,201	5,329	-,195	-1,351	,181	-17,851	3,449	,592	1,689
	Role_reversal	10,674	5,097	,410	2,094	,040	,488	20,861	,321	3,114
	Coparenting_support	-13,592	5,256	-,467	-2,586	,012	-24,095	-3,089	,377	2,649

a. Dependent Variable: HRV_baseline

Multiple linear regression 5

Predictors: FCAAS scales (+ control variables: age and sex of adolescent)

Outcome: HRV_reactivity

Variables Entered/Removed[a]

Model	Variables Entered	Variables Removed	Method
1	Ado_sex, Ado_age[b]	.	Enter
2	Postures_gazes, Mentalization, Adolescent_autonomy, Turn_taking, Conflict_resolution, Affective_climate, Autonomy_promotion[b]	.	Enter
3	Mutual_respect_for_coparenting_roles, Coparenting_support, Role_reversal[b]	.	Enter

a. Dependent Variable: HRV_reactivity

b. All requested variables entered.

Model Summary

Model	R	R Square	Adjusted R Square	Std. Error of the Estimate	Change Statistics				
					R Square Change	F Change	df1	df2	Sig. F Change
1	,194[a]	,037	,011	19,55872	,037	1,401	2	72	,253
2	,310[b]	,096	-,029	19,94634	,059	,604	7	65	,750
3	,491[c]	,241	,094	18,71314	,145	3,950	3	62	,012

a. Predictors: (Constant), Ado_sex, Ado_age

b. Predictors: (Constant), Ado_sex, Ado_age, Postures_gazes, Mentalization, Adolescent_autonomy, Turn_taking, Conflict_resolution, Affective_climate, Autonomy_promotion

c. Predictors: (Constant), Ado_sex, Ado_age, Postures_gazes, Mentalization, Adolescent_autonomy, Turn_taking, Conflict_resolution, Affective_climate, Autonomy_promotion, Mutual_respect_for_coparenting_roles, Coparenting_support, Role_reversal

ANOVA[a]

Model		Sum of Squares	df	Mean Square	F	Sig.
1	Regression	1071,693	2	535,847	1,401	,253[b]
	Residual	27543,142	72	382,544		
	Total	28614,836	74			
2	Regression	2754,166	9	306,018	,769	,645[c]
	Residual	25860,670	65	397,856		
	Total	28614,836	74			
3	Regression	6903,581	12	575,298	1,643	,103[d]
	Residual	21711,255	62	350,182		
	Total	28614,836	74			

a. Dependent Variable: HRV_reactivity

b. Predictors: (Constant), Ado_sex, Ado_age

c. Predictors: (Constant), Ado_sex, Ado_age, Postures_gazes, Mentalization, Adolescent_autonomy, Turn_taking, Conflict_resolution, Affective_climate, Autonomy_promotion

d. Predictors: (Constant), Ado_sex, Ado_age, Postures_gazes, Mentalization, Adolescent_autonomy, Turn_taking, Conflict_resolution, Affective_climate, Autonomy_promotion, Mutual_respect_for_coparenting_roles, Coparenting_support, Role_reversal

Coefficients[a]

Model		Unstandardized Coefficients		Standardized Coefficients	t	Sig.	95.0% Confidence Interval for B		Collinearity Statistics	
		B	Std. Error	Beta			Lower Bound	Upper Bound	Tolerance	VIF
1	(Constant)	25,814	30,134		,857	,394	-34,258	85,886		
	Ado_age	-1,341	2,468	-,063	-,543	,589	-6,262	3,579	,994	1,006
	Ado_sex	7,059	4,598	,178	1,535	,129	-2,107	16,224	,994	1,006
2	(Constant)	21,159	32,761		,646	,521	-44,269	86,586		
	Ado_age	-1,347	2,702	-,063	-,499	,620	-6,743	4,048	,863	1,159
	Ado_sex	4,590	5,108	,116	,899	,372	-5,612	14,792	,837	1,194
	Postures_gazes	-1,762	2,663	-,088	-,662	,510	-7,080	3,555	,779	1,283
	Turn_taking	1,867	3,090	,096	,604	,548	-4,305	8,038	,548	1,825
	Conflict_resolution	3,646	3,465	,194	1,052	,297	-3,273	10,565	,411	2,432
	Affective_climate	-1,082	3,225	-,067	-,335	,738	-7,523	5,359	,352	2,839
	Mentalization	-3,520	2,825	-,227	-1,246	,217	-9,161	2,122	,418	2,393
	Autonomy_promotion	2,224	3,501	,137	,635	,528	-4,769	9,216	,298	3,354
	Adolescent_autonomy	-,407	2,388	-,025	-,170	,865	-5,176	4,363	,621	1,609
3	(Constant)	66,371	34,056		1,949	,056	-1,706	134,449		
	Ado_age	-2,941	2,659	-,138	-1,106	,273	-8,256	2,374	,784	1,275
	Ado_sex	4,913	4,887	,124	1,005	,319	-4,856	14,681	,805	1,242
	Postures_gazes	-3,183	2,569	-,160	-1,239	,220	-8,318	1,953	,737	1,357
	Turn_taking	3,242	2,991	,167	1,084	,283	-2,736	9,220	,515	1,942
	Conflict_resolution	5,719	3,567	,304	1,603	,114	-1,411	12,849	,341	2,929
	Affective_climate	,466	3,078	,029	,152	,880	-5,686	6,619	,340	2,937
	Mentalization	-3,304	2,730	-,213	-1,210	,231	-8,761	2,153	,394	2,539
	Autonomy_promotion	,742	3,376	,046	,220	,827	-6,006	7,489	,282	3,543
	Adolescent_autonomy	-1,427	2,333	-,089	-,612	,543	-6,089	3,236	,573	1,744
	Mutual_respect_for_coparenting_roles	-6,012	2,984	-,290	-2,015	,048	-11,976	-,047	,592	1,689
	Role_reversal	6,781	2,854	,464	2,376	,021	1,076	12,485	,321	3,114
	Coparenting_support	-7,697	2,943	-,471	-2,616	,011	-13,579	-1,815	,377	2,649

a. Dependent Variable: HRV_reactivity

Multiple linear regression 6

Predictors: FCAAS scales (+ control variables: age and sex of adolescent)

Outcome: HRV_recovery

Variables Entered/Removed[a]

Model	Variables Entered	Variables Removed	Method
1	Ado_sex, Ado_age[b]	.	Enter
2	Postures_gazes, Mentalization, Adolescent_autonomy, Turn_taking, Conflict_resolution, Affective_climate, Autonomy_promotion[b]	.	Enter
3	Mutual_respect_for_coparenting_roles, Coparenting_support, Role_reversal[b]	.	Enter

a. Dependent Variable: HRV_recovery

b. All requested variables entered.

Model Summary

Model	R	R Square	Adjusted R Square	Std. Error of the Estimate	Change Statistics				
					R Square Change	F Change	df1	df2	Sig. F Change
1	,176[a]	,031	,003	19,22880	,031	1,114	2	70	,334
2	,272[b]	,074	-,059	19,81474	,043	,417	7	63	,888
3	,428[c]	,183	,020	19,06488	,110	2,684	3	60	,055

a. Predictors: (Constant), Ado_sex, Ado_age

b. Predictors: (Constant), Ado_sex, Ado_age, Postures_gazes, Mentalization, Adolescent_autonomy, Turn_taking, Conflict_resolution, Affective_climate, Autonomy_promotion

c. Predictors: (Constant), Ado_sex, Ado_age, Postures_gazes, Mentalization, Adolescent_autonomy, Turn_taking, Conflict_resolution, Affective_climate, Autonomy_promotion, Mutual_respect_for_coparenting_roles, Coparenting_support, Role_reversal

ANOVA[a]

Model		Sum of Squares	df	Mean Square	F	Sig.
1	Regression	823,448	2	411,724	1,114	,334[b]
	Residual	25882,269	70	369,747		
	Total	26705,718	72			
2	Regression	1970,408	9	218,934	,558	,826[c]
	Residual	24735,310	63	392,624		
	Total	26705,718	72			
3	Regression	4897,540	12	408,128	1,123	,359[d]
	Residual	21808,178	60	363,470		
	Total	26705,718	72			

a. Dependent Variable: HRV_recovery

b. Predictors: (Constant), Ado_sex, Ado_age

c. Predictors: (Constant), Ado_sex, Ado_age, Postures_gazes, Mentalization, Adolescent_autonomy, Turn_taking, Conflict_resolution, Affective_climate, Autonomy_promotion

d. Predictors: (Constant), Ado_sex, Ado_age, Postures_gazes, Mentalization, Adolescent_autonomy, Turn_taking, Conflict_resolution, Affective_climate, Autonomy_promotion, Mutual_respect_for_coparenting_roles, Coparenting_support, Role_reversal

Coefficients[a]

Model		Unstandardized Coefficients		Standardized Coefficients	t	Sig.	95.0% Confidence Interval for B		Collinearity Statistics	
		B	Std. Error	Beta			Lower Bound	Upper Bound	Tolerance	VIF
1	(Constant)	32,924	30,035		1,096	,277	-26,979	92,826		
	Ado_age	-2,254	2,460	-,108	-,916	,363	-7,161	2,653	,994	1,006
	Ado_sex	5,050	4,583	,130	1,102	,274	-4,090	14,189	,994	1,006
2	(Constant)	25,173	32,993		,763	,448	-40,758	91,105		
	Ado_age	-1,474	2,721	-,071	-,542	,590	-6,911	3,963	,863	1,159
	Ado_sex	4,608	5,145	,119	,896	,374	-5,672	14,889	,837	1,194
	Postures_gazes	,093	2,682	,005	,035	,972	-5,266	5,452	,779	1,283
	Turn_taking	1,727	3,112	,091	,555	,581	-4,492	7,946	,548	1,825
	Conflict_resolution	1,301	3,489	,070	,373	,711	-5,672	8,273	,411	2,432
	Affective_climate	-1,057	3,248	-,067	-,326	,746	-7,548	5,433	,352	2,839
	Mentalization	-2,984	2,845	-,197	-1,049	,298	-8,669	2,701	,418	2,393
	Autonomy_promotion	2,024	3,526	,127	,574	,568	-5,023	9,070	,298	3,354
	Adolescent_autonomy	-1,716	2,405	-,110	-,714	,478	-6,523	3,090	,621	1,609
3	(Constant)	66,280	35,175		1,884	,064	-4,081	136,640		
	Ado_age	-2,887	2,746	-,139	-1,051	,297	-8,380	2,606	,784	1,275
	Ado_sex	4,519	5,047	,116	,895	,374	-5,577	14,615	,805	1,242
	Postures_gazes	-1,328	2,654	-,068	-,501	,619	-6,636	3,980	,737	1,357
	Turn_taking	2,558	3,089	,135	,828	,411	-3,620	8,737	,515	1,942
	Conflict_resolution	2,694	3,684	,146	,731	,467	-4,675	10,063	,341	2,929
	Affective_climate	,097	3,179	,006	,030	,976	-6,263	6,456	,340	2,937
	Mentalization	-3,139	2,820	-,207	-1,113	,270	-8,779	2,501	,394	2,539
	Autonomy_promotion	1,003	3,487	,063	,288	,775	-5,971	7,977	,282	3,543
	Adolescent_autonomy	-2,521	2,409	-,161	-1,046	,300	-7,340	2,298	,573	1,744
	Mutual_respect_for_coparenting_roles	-6,061	3,082	-,298	-1,967	,054	-12,225	,104	,592	1,689
	Role_reversal	6,279	2,948	,439	2,130	,037	,383	12,175	,321	3,114
	Coparenting_support	-5,522	3,039	-,345	-1,817	,074	-11,602	,557	,377	2,649

a. Dependent Variable: HRV_recovery
